# SR9009 induces a REV-ERB dependent anti-small-cell lung cancer effect through inhibition of autophagy

**DOI:** 10.7150/thno.42478

**Published:** 2020-03-15

**Authors:** Weitao Shen, Wei Zhang, Weilin Ye, Haihong Wang, Qingxi Zhang, Jie Shen, Qingsha Hong, Xiang Li, Ge Wen, Ting Wei, Jian Zhang

**Affiliations:** 1 Department of Oncology, Zhujiang Hospital, Southern Medical University, 253 Industrial Avenue, Guangzhou, 510282, Guangdong, People's Republic of China.; 2 Department of Histology and Embryology, School of Basic Medical Sciences, Southern Medical University, Guangzhou, 510515, Guangdong, People's Republic of China.; 3 The Second School of Clinical Medicine, Southern Medical University, No.1023, South Shatai Road, Baiyun District, Guangzhou, 510515, Guangdong, People's Republic of China.; 4 Department of Obstetrics & Gynecology, Nanfang Hospital, Southern Medical University, Guangzhou, 510515, People's Republic of China.; 5 Division of Laboratory Medicine, Zhujiang Hospital, Southern Medical University, 253 Industrial Avenue, Guangzhou, 510282, Guangdong, People's Republic of China.; 6 Department of Research, Bioillus technology Co.Ltd, Guangzhou 510631, Guangdong, People's Republic of China.

**Keywords:** small cell lung cancer, circadian clock component, SR9009, REV-ERB, autophagy

## Abstract

**Rationale**: The circadian clock coordinates cell proliferation and metabolism and impacts the progression of some diseases, particularly cancer. Pharmacological modulation of the circadian machinery may be an effective therapeutic approach for treating cancer. SR9009 is a specific synthetic agonist of the REV-ERBs, essential circadian clock components. However, the potential efficacy and antitumor mechanism of this drug in small-cell lung cancer (SCLC) remains poorly understood.

**Methods**: Here, we used chemosensitive cells (H69 and H446) and the corresponding chemoresistant cells (H69AR and H446DDP) to assess the efficacy of the REV-ERB agonist SR9009 for the treatment of SCLC *in vitro* and further validated the antitumor effect in subcutaneous tumor models of SCLC. Then, we determined whether REV-ERBα was correlated with the anti-SCLC effect of SR9009. Chromatin immunoprecipitation (ChIP) sequencing assays were conducted to identify potential DNA sequences directly regulated by REV-ERBα. Autophagy regulation by REV-ERBα and its possible mechanism in SR9009-based SCLC therapy were analyzed.

**Results**: Here, we showed that the REV-ERB agonist SR9009 is specifically lethal to both chemosensitive and chemoresistant SCLC cells. REV-ERBα was involved in the antitumor effect of SR9009 in SCLC. The core autophagy gene Atg5 was identified as a direct downstream target of REV-ERBα and was suppressed by the REV-ERB agonist SR9009 in SCLC. Furthermore, the interaction of REV-ERBα with this autophagy gene impaired autophagy activity, leading to SR9009 cytotoxicity in SCLC cells.

**Principal conclusions**: Our study provided a novel viewpoint indicating that the REV-ERB agonist SR9009 could be a novel and promising therapeutic strategy in first- or second-line SCLC treatment. The anti-SCLC effect of SR9009 is mediated by REV-ERB dependent suppression of autophagy via direct repression of the autophagy gene Atg5.

## Introduction

Small cell lung cancer (SCLC) is a fairly aggressive malignant tumor that affects an estimated 280,000 individuals per year worldwide, making it the seventh most common cause of cancer mortality 1,2. SCLC results in poor survival: patients diagnosed with early-stage SCLC have a median survival time of 15-20 months post-diagnosis, while patients with more widely disseminated late-stage SCLC have a median survival of only 9-12 months 3. Even when SCLC is diagnosed prior to metastasis, most patients diagnosed with early-stage disease will experience tumor recurrence and death within the first two years after diagnosis. The current standard first-line treatment for SCLC consists of radiotherapy and platinum-based doublet chemotherapy with etoposide (VP-16) in combination with a platinating drug 4. However, more than 75% of patients are diagnosed at an advanced, irreversible stage when treatment options are limited, multidrug resistance to chemotherapy rapidly emerges, and dose-limiting toxicities become problematic 5. Thus, more effective therapeutic strategies for SCLC are urgently needed.

The cell-autonomous circadian clock regulates the complicated network of physiological processes and affects pathological process 6-9. The importance of proper clock maintenance is highlighted by linkages between circadian desynchrony and a wide range of diseases, especially cancers 10,11. Considering that the circadian clock can effectively modulate many pathways important for tumorigenesis and progression, pharmacological regulation of circadian rhythm components may offer promising effective antitumor approaches.

The haem-binding circadian clock components REV-ERBs (REV-ERBα/NR1D1 and REVERBβ/NR1D2) function as transcriptional repressors involved in aspects of tumorigenesis, including metabolism and proliferation 12-14. Similarly to other nuclear receptors, REV-ERBs activity is modulated upon binding with a ligand (the tetrapyrrole haem). In attempts to pharmacologically target REV-ERBs, SR9009, a specific agonist of pyrrole derivatives, was developed. SR9009, a synthetic REV-ERB agonist, is a promising pharmaceutical agent, and its efficacy in treating several conditions has been studied. These conditions include metabolic diseases such as obesity, bipolar, anxiety and depressive disorders; and as an alternative medication for sleep cycle disturbances 15. Recently, SR9009 has been reported to exert a cytotoxic effect on cancer cells, including leukemia and melanoma cells, *in vitro* and to exhibit a remarkable antitumor effect in a xenograft animal model of glioblastoma *in vivo* 16*.* However, the anticancer effect on either the chemosensitivity or chemoresistance of SCLC has never been explored. As REV-ERBs lack the canonical NR activation domain and act as negative transcription factors, it is also critical to elucidate REV-ERB-regulated genes and REV-ERB-modulated mechanisms to identify the cytotoxic effect of the REV-ERB agonist SR9009 on SCLC.

Woldt E et al found that REV-ERBs could regulate autophagy activity to induce corresponding biological effects 17. Autophagy is a natural physiologic process that maintains cellular homeostasis via the degradation of unnecessary or dysfunctional cellular components, although it acts as a double-edged sword in cancer 18,19. Most studies have suggested that autophagy can be a target for cancer therapy 20-23. However, to date, the concrete molecular mechanisms by which the circadian clock components REV-ERBs and their agonist SR9009 regulate autophagy and the corresponding rhythmic activity in SCLC are unclear. Thus, ascertaining whether the potential antitumor effects of SR9009 are mediated by autophagy or other pathways is critical for developing future clinical applications.

In this paper, we report that the REV-ERB agonist SR9009 induced marked antitumor effects on both chemosensitive (H69 and H446) and the corresponding chemoresistant (H69AR and H446DDP) SCLC cells. In addition, SR9009 induced caspase-dependent apoptosis. Notably, we demonstrated that SR9009 impaired SCLC growth and attained good efficacy when combined with chemotherapy* in vivo*. Furthermore, we identified that REV-ERBα was involved in the antitumor effect of SR9009 in SCLC. Our chromatin immunoprecipitation (ChIP) assays showed that Rev-erbα binds directly to the core autophagy gene Atg5 and suppresses it. Then, we revealed that the REV-ERB agonist SR9009 negatively regulated autophagy. These discoveries may explain the antitumor mechanism of SR9009 through the regulation of autophagy in SCLC. Therefore, our study suggests a novel approach for SCLC therapy and provides new ideas for first- or second-line SCLC clinical treatment in the future.

## Results

### The REV-ERB agonist SR9009 exhibited potent antitumor effect to both chemosensitive and chemoresistant SCLC cells *in vitro*

**Figure 1:**
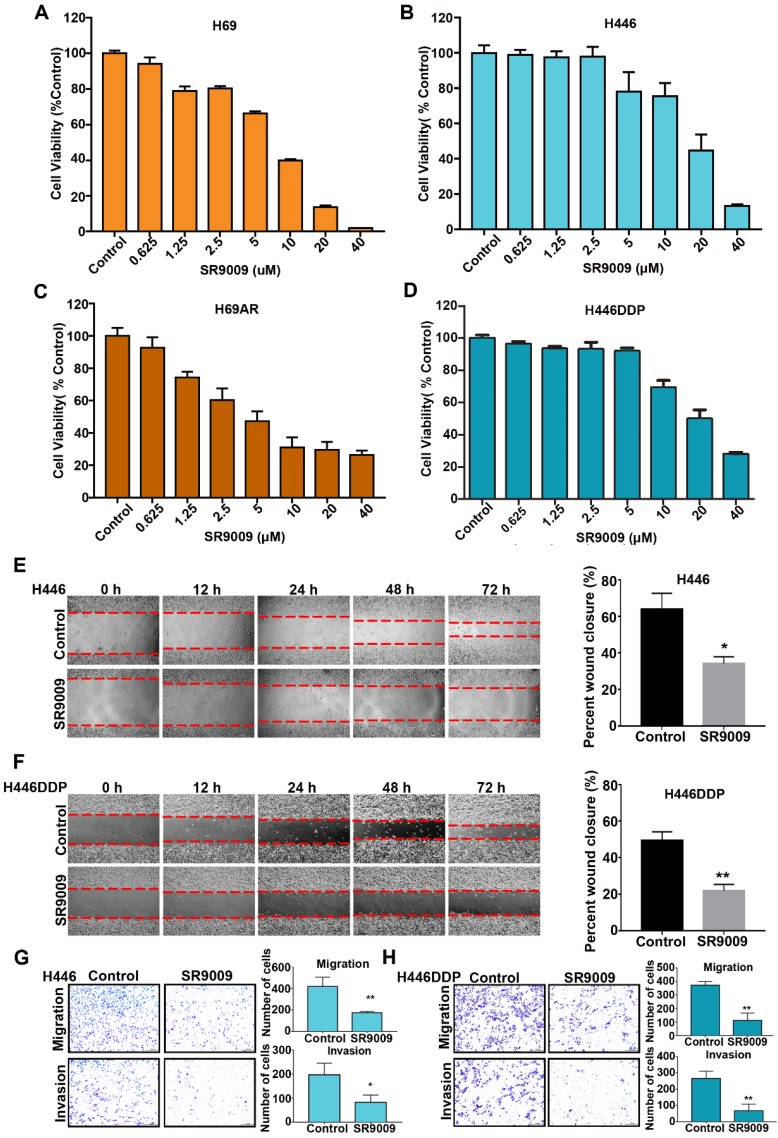
** REV-ERBs agonists SR9009 is lethal in both Chemosensitive cells and their corresponding chemotherapy-resistant cells. (A and B)** Chemosensitive cells (H69 and H446) were incubated for 72 h in the presence or absence of different concentrations of SR9009. **(C and D)** chemotherapy-resistant cells (H69AR and H446DDP) were treated with different concentrations of SR9009 for 72 h. **(A-D)** The data are presented as means ± S.D. of three independent experiments. **(E and F)** Cell migration ability was studied by scratch-wound assay. After being washed by PBS, the wound areas were photographed at 0 h, 12 h, 24 h, 48 h and 72 h. The percent wound closure = (the width of the wound at 72 h - the width of the wound at 0 h)/the width of the wound at 0 h. The data are presented as means ± S.D. of three independent experiments. *P < 0.05; **P < 0.01. **(G and H)** Transwell assays were performed to investigate changes in cell migration and invasiveness. Error bars, mean ± SD from three independent experiments. *P < 0.05; **P < 0.01.

First, we investigated whether the REV-ERB agonist SR9009 exhibited cytotoxicity to SCLC cells *in vitro*. Chemosensitive cells (H69 and H446) and their corresponding chemoresistant cells (H69AR and H446DDP) were used in our experiment. SCLC cells were treated with increasing concentrations of SR9009 for 72 h. As shown in Figure 1A-D, significant dose-dependent cytotoxicity was induced by SR9009 in both chemosensitive cells and their corresponding chemoresistant cells. These preliminary data suggest that SR9009 could be an effective drug for first- or second-line treatment of SCLC. In addition, we also evaluated the effect of SR9009 on cell migration and invasion which were significant characteristics of cancer progression. Wound-healing assays and transwell migration assays showed that SR9009 significantly decreased cell migratory behaviors, and transwell matrigel invasion assays demonstrated that the invasive behaviors of cells could be inhibited after treated by SR9009 (Figure 1E-H). These results suggested that SR9009 could alleviate the cancer progression.

### SR9009 induced caspase-dependent apoptosis in SCLC cells

**Figure 2:**
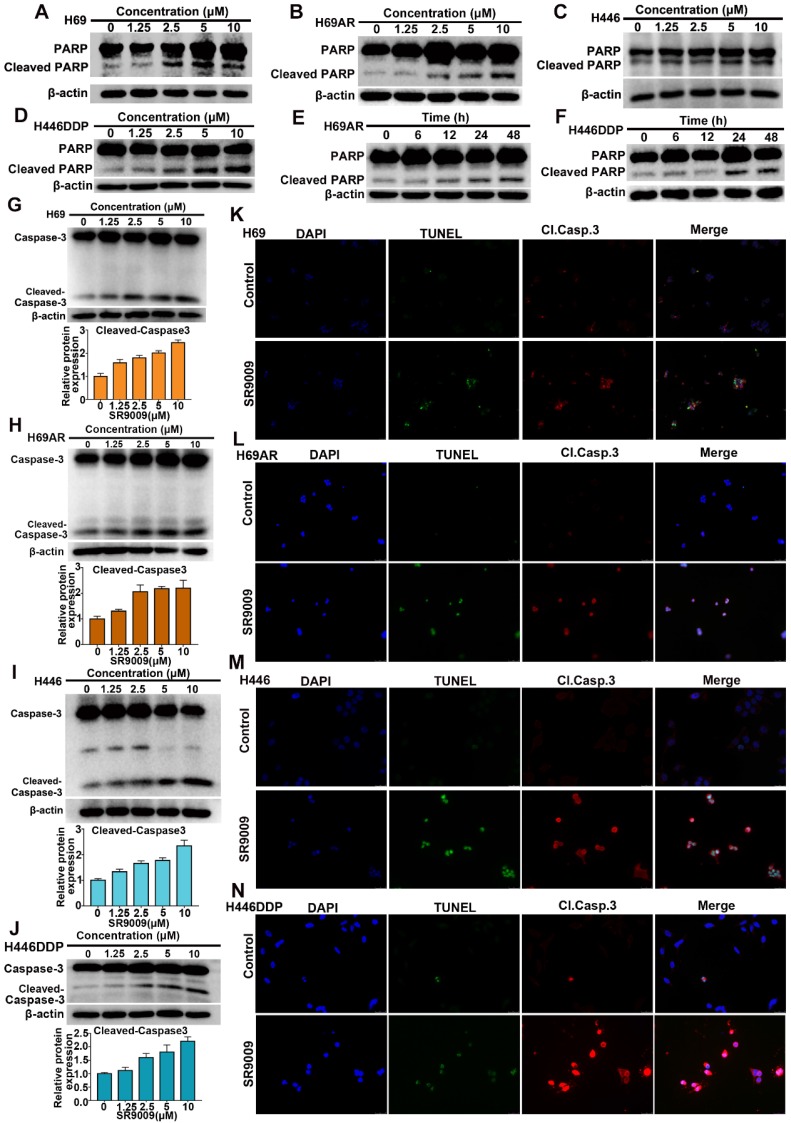
** SR9009 induced remarkable apoptosis in SCLC cells. (A-D)** SCLC cells were dose-dependently treated with SR9009 for 48 h. **(E-F)** H69AR and H446DDP cells were time dependently treated with 10 µM SR9009. **(G-J)** SCLC cells were concentration-dependently treated with SR9009 for 48 h. Densitometric values were quantified using the ImageJ software and normalized to control. The values of control were set to 1. The data are presented as means ± SD of three independent experiments. **(K-N)** Induction of apoptosis by indicated concentration of SR9009 is shown by cleaved caspase 3 and TUNEL staining for 72 h in SCLC cells (K, 10µM; L, 5µM; M, 20µM; N, 10µM). A typical result from 3 independent experiments is presented.

Because many antitumor drugs induce cell death through apoptosis, we determined whether apoptosis could be triggered by SR9009 in SCLC cells. As shown in Figure 2A-J and Figure S1A-F, western blot assays demonstrated that the apoptotic proteins PARP and caspase 3 were activated after treatment with SR9009. The impairment of SCLC cell viability upon treatment with SR9009 was partially due to the induction of apoptosis, as evaluated by immunofluorescence assays for cleaved caspase 3 and terminal deoxynucleotidyl transferase (TdT) dUTP nick end labeling (TUNEL) assays (Figure 2K-N).

**Figure 3:**
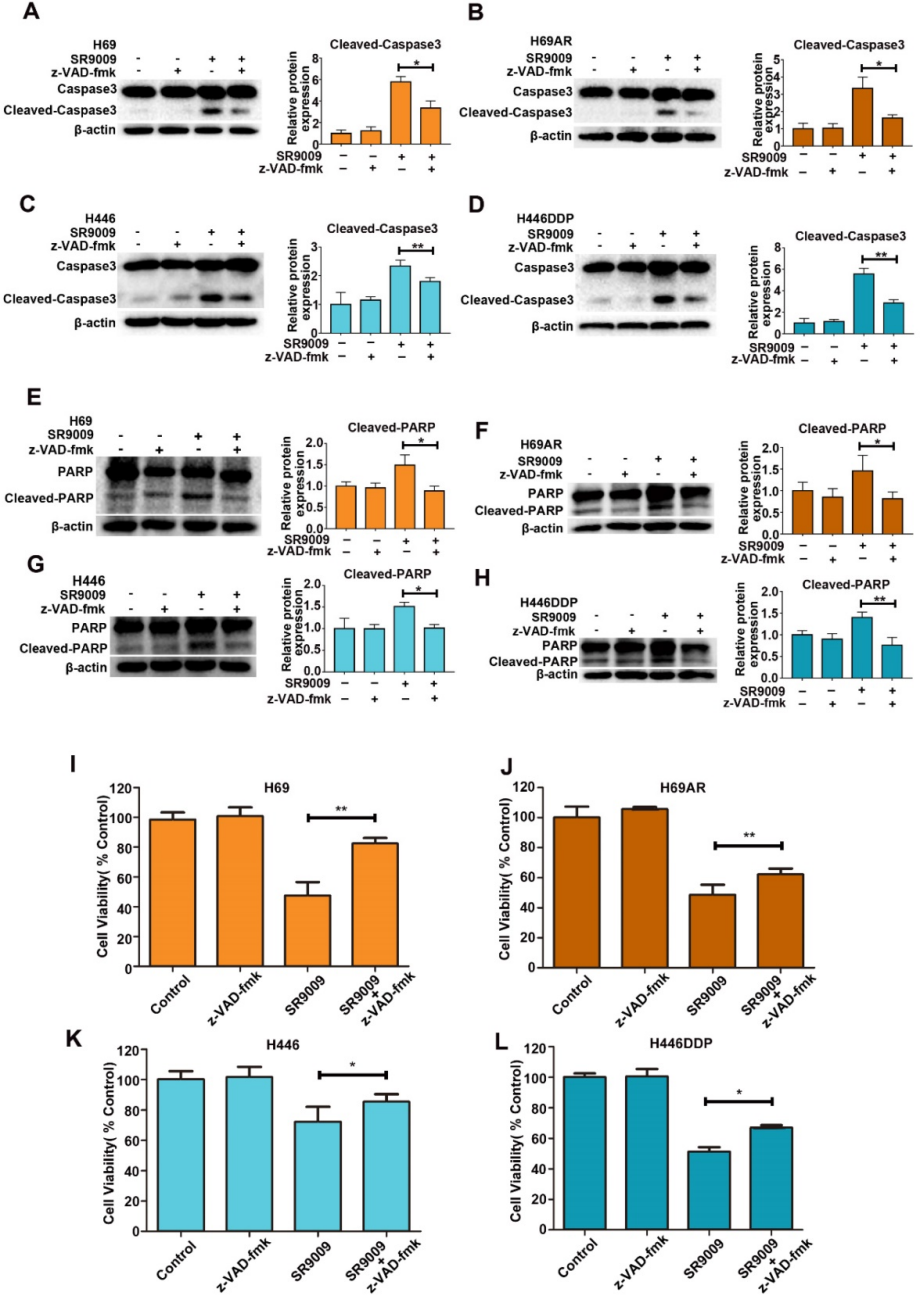
** SR9009-induced apoptosis was caspase-dependent in SCLC cells. (A-H)** SCLC cells were incubated with indicated concentration of SR9009 in the presence or absence of 20 µM z-VAD-fmk for 48 h (A and E, 10µM; B and F, 5µM; C and G, 20µM; D and H, 10µM). Densitometric values were quantified using the ImageJ software and normalized to control. The values of control were set to 1. The data are presented as means ± SD of three independent experiments. *P < 0.05; **P < 0.01. **(I-L)** SR9009-induced cytotoxicity could be partially rescued by 20 µM z-VAD-fmk for 72 h (concentration of SR9009: I, 10µM; J, 5µM; K, 20µM; L, 10µM). Results were expressed as mean ± S.D. (n = 3) and analyzed by Student's t-test (two-tailed), *P< 0.05; **P< 0.01.

To identify whether SR9009-induced apoptosis was caspase-dependent, we utilized a general caspase family inhibitor, benzyloxycarbonyl Val-Ala-Asp (O-methyl)-fluoro-methylketone (z-VAD-fmk), in the follow-up experiment. When SCLC cells were pretreated with z-VAD-fmk, the level of cleaved caspase 3 significantly decreased (Figure 3A-D). The level of cleaved PARP also decreased (Figure 3E-H). Furthermore, cytotoxicity induced by SR9009 could be partially rescued by z-VAD-fmk (Figure 3I-L). Together, these results indicate that caspase-dependent apoptosis can be induced by SR9009 in SCLC cells.

### SR9009 exhibited antitumor efficacy in the SCLC subcutaneous tumor model

**Figure 4:**
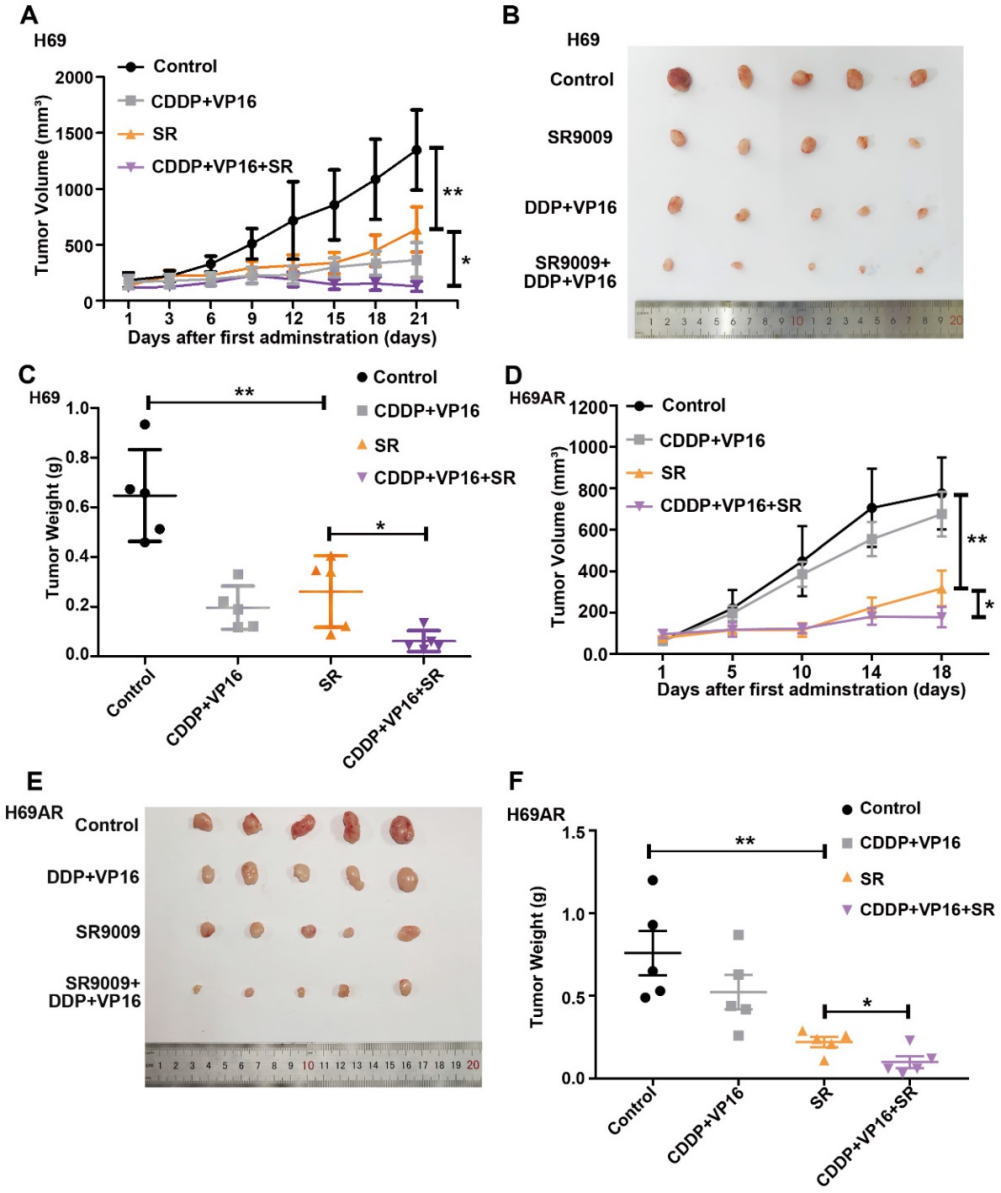
** SR9009 exhibited remarkable anti-tumor effect on SCLC* in vivo.* (A and D)** Tumor growth between different groups was measured and calculated by an ellipsoid volume formula (length × width^2^/2). (*P<0.05, **P<0.01) **(B and E)** Effect of SR9009, chemotherapy (CDDP and VP-16), or combination of SR9009 and chemotherapy on tumor growth using subcutaneous SCLC models. **(C and F)** The tumor weight of different groups was measured at the last day of animal experiments (*P<0.05, **P<0.01).

To determine whether SR9009 exhibits antitumor effects *in vivo*, subcutaneous tumor models were successfully established in nude mice with both chemosensitive and chemoresistant SCLC cells. Tumor-bearing mice were intraperitoneally administered either SR9009, chemotherapeutic drugs (cisplatin (CDDP) and VP-16), or a combination. Consistent with the *in vitro* observations discussed above, SR9009 treatment led to marked tumor growth inhibition in both the chemosensitive and chemoresistant tumor models (Figure 4A-F).

Interestingly, tumor growth was also significantly different between the SR9009 group vs the CDDP+VP16+SR9009 group *in vivo* (Figure 4A-F). We further evaluated relative drug sensitivity of chemoresistant SCLC cells after pretreated with SR9009. Consistent with the above results, pretreatment with SR9009 could sensitize the resistant cells to chemotherapy (Figure S2), which extended the applications of SR9009 in the treatment of SCLC.

Thus, SR9009 exhibited substantial antitumor effects against SCLC independent of the chemoresistance status *in vivo*, indicating the considerable and broad prospects of SR9009 in first- or second-line SCLC treatment.

### REV-ERBα was involved in the antitumor effect of SR9009 in SCLC

**Figure 5:**
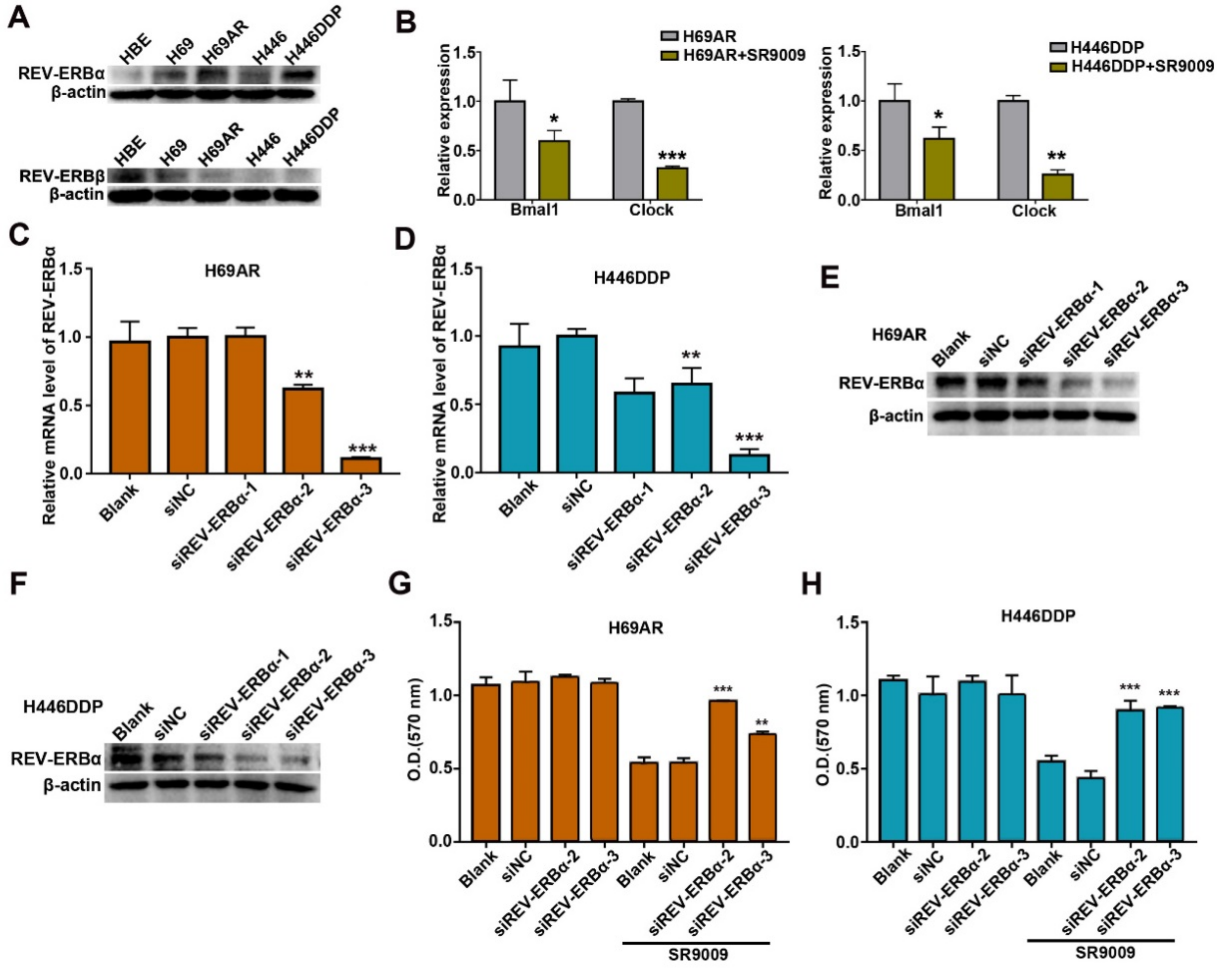
** REV-ERBα was involved in the anti-SCLC effect of SR9009. (A)** The protein expression of REV-ERB was measured by western blot analysis in HBE cells and two pairs of chemosensitive and chemoresistant SCLC cell lines. A typical result from 3 independent experiments is presented. **(B)** QRT-PCR of Bmal1 and Clock in the presence or absence of 10 µM SR9009. The data are presented as means ± SD of three independent experiments. *P < 0.05; **P < 0.01. **(C and F)** QRT-PCR and Western blot analysis of REV-ERBα in H69AR and H446DDP cells transfected with si-REV-ERBα or negative control vector. The data are presented as means ± SD of three independent experiments. *P < 0.05, **P < 0.01 versus to the group of cells transfected with negative control vector. **(G)** CCK-8 assays showed the effect of downregulation of REV-ERBα on cytotoxicity induced by SR9009 (10 µm) in H69AR cells. **(H)** CCK-8 assays showed the effect of downregulation of REV-ERBα on cytotoxicity induced by SR9009 (10 µm) in H446DDP cells. **(G and H)** The data are presented as means ± SD of three independent experiments. **P < 0.01, ***P < 0.001 versus to the group of cells transfected with negative control vector.

Previous studies demonstrated that the pyrrole derivative SR9009 can pharmacologically activate REV-ERBs. To ascertain whether the anti-SCLC effects of SR9009 are mediated by REV-ERBs, we utilized normal human bronchial epithelial (HBE) cells as control cells. The basal expression of REV-ERBα and REV-ERBβ was examined in HBE cells and two pairs of chemosensitive and chemoresistant SCLC cell lines. Compared with normal control cells, SCLC cells expressed fairly high levels of the REV-ERBα protein but low expression of the REV-ERBβ protein (Figure 5A). Then, we evaluated whether the protein expression of REV-ERBα could be increased after treated by SR9009. However, results showed that the protein expression of REV-ERBα was not significantly increased following treatment with SR9009 (Figure S3A and B). To assessing efficiency of REV-ERBs activation by SR9009, we quantified mRNA abundance of some of their targets, such as Bmal1 and Clock. The results showed that mRNA abundance of Bmal1 and Clock was remarkably decreased after treatment with SR9009 (Figure 5B).

Thus, we speculated that SR9009 interacted with REV-ERB through pharmacological activation in post-translational manner, in accordance with the results of previous studies.

To further identify the target by which SR9009 induces antitumor effect in SCLC, REV-ERBα, highly expressed in H69AR and H446DDP cells, was knocked down by small interfering RNA (siRNA) (Figure 5C-F). The anticancer activity of SR9009 was abolished following downregulation of REV-ERBα (Figure 5G and H). Moreover, to exclude the possibility of non-specific binding and signaling, we evaluated the effect of SR9009 on normal human bronchial epithelial (HBE) cells, and results suggested that the concentration of SR9009 used in this study couldn't impair the normal human bronchial epithelial (HBE) cells (Figure S3C).

These results suggest that REV-ERBα was involved in the antitumor effect of SR9009 in SCLC.

### REV-ERBα repressed the core autophagy gene Atg5 via direct binding to the Atg5 gene promoter

**Figure 6:**
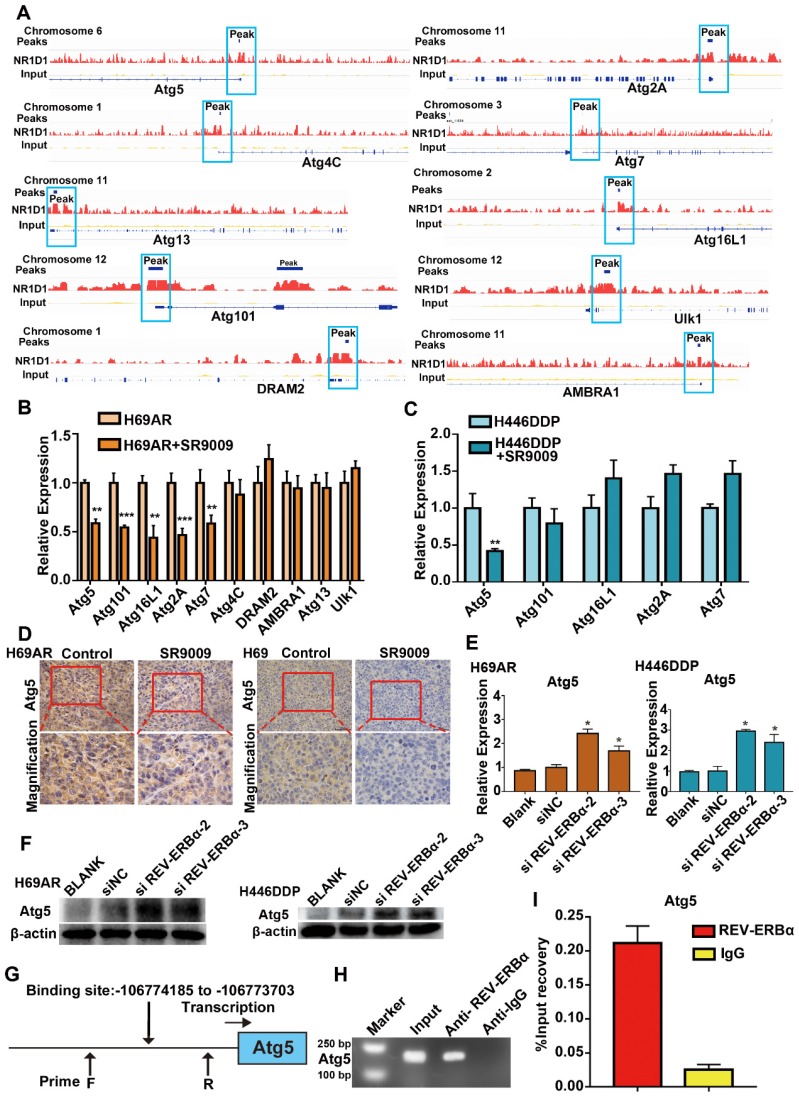
** Core autophagy gene Atg5 was novel and functional REV-ERBα target in SCLC. (A)** Analyses of ChIP-sequence data indicated that REV-ERBs peaks were present in core autophagy genes Atg5, Atg101, Atg16L1, Atg2A, Atg7, Atg4C, DRAM2, AMBRA1, Atg13 and Ulk1 (false discovery rate ≤ 0.05). **(B)** QRT-PCR analysis of autophagy gene Atg5, Atg101, Atg16L1, Atg2A, Atg7, Atg4C, DRAM2, AMBRA1, Atg13 and Ulk1 in H69AR cells. The values of relative control were set to 1. The data are presented as means ± SD of three independent experiments. **P < 0.01; ***P < 0.001. **(C)** QRT-PCR analysis of autophagy gene Atg5, Atg101, Atg16L1, Atg2A and Atg7 in H446DDP cells. The values of relative control were set to 1. The data are presented as means ± SD of three independent experiments. **P < 0.01.** (D)** Representative IHC staining of autophagy associate protein Atg5 in subcutaneous xenografts of SR9009-treated group or vehicle group. **(E and F)** Autophagy core genes Atg5 was upregulated in cells with depletion of REV-ERBα by siRNA. The values of relative control were set to 1. The data are presented as means ± SD of three independent experiments. *P < 0.05. **(G)** Predicted REV-ERBα binding site and the qPCR primer location in the Atg5 promoter region. **(H and I)** REV-ERBα ChIP-qPCR assessing REV-ERBα enrichment at the Atg5 promoter. The data are presented as means ± SD of three independent experiments.

REV-ERBα can functionally repress gene transcription by competing for binding to the promoter regions of its downstream genes. Published data indicate that REV-ERBα directly regulates the autophagy gene ULK1 to impair autophagy activity in zebrafish; however, whether this mechanism is active in SCLC remains unknown 24. The results of our ChIP sequencing (ChIP-seq) assays revealed the presence of peaks in the core autophagy genes Atg5, Atg2A, Atg4C, Atg7, Atg13, Atg16L1, Atg101, AMBRA1, DRAM2 and Ulk1, suggesting that REV-ERBα may directly bind to the promoters of autophagy genes in SCLC cells (Figure 6A). Then, we applied the REV-ERBα agonist SR9009 to identify the autophagy genes with repressed expression due to the binding of REV-ERBα to the promoter. The expression of the Atg5 gene was most markedly blocked after treatment with the REV-ERBα agonist SR9009 in both H69AR and H446DDP cells (Figure 6B and C). Furthermore, we examined the expression of Atg5 in the abovementioned SCLC subcutaneous tumor models through immunohistochemistry (IHC), which also showed that Atg5 expression was blocked in the SR9009-treated group (Figure 6D), whereas the expression of this gene was induced following depletion of REV-ERBα by siRNA(Figure 6E and F). To further investigate the possible role of REV-ERBα in the regulation of Atg5, we predicted the REV-ERBα binding site in the Atg5 promoter region based on the UCSC database and analysis of the Atg5 peaks in the ChIP-seq data (Figure 6G). We next conducted ChIP quantitative PCR (ChIP-qPCR) assays. The subsequent REV-ERBα ChIP-qPCR assay showed that REV-ERBα was enriched in the Atg5 promoter, suggesting that REV-ERBα might repress Atg5 expression through binding to its promoter and inhibiting promoter activity (Figure 6H and I). Collectively, these results demonstrate that the core autophagy gene Atg5 is a direct target of REV-ERBα and is negatively regulated by REV-ERBα in SCLC cells.

### Autophagy was inhibited by the REV-ERB agonist SR9009 in the treatment of SCLC

As we discovered above, REV-ERBα directly negatively regulates the autophagy gene Atg5. These observations prompted us to investigate whether autophagy defects are involved in the anti-SCLC activity of the REV-ERBα agonist SR9009.

**Figure 7:**
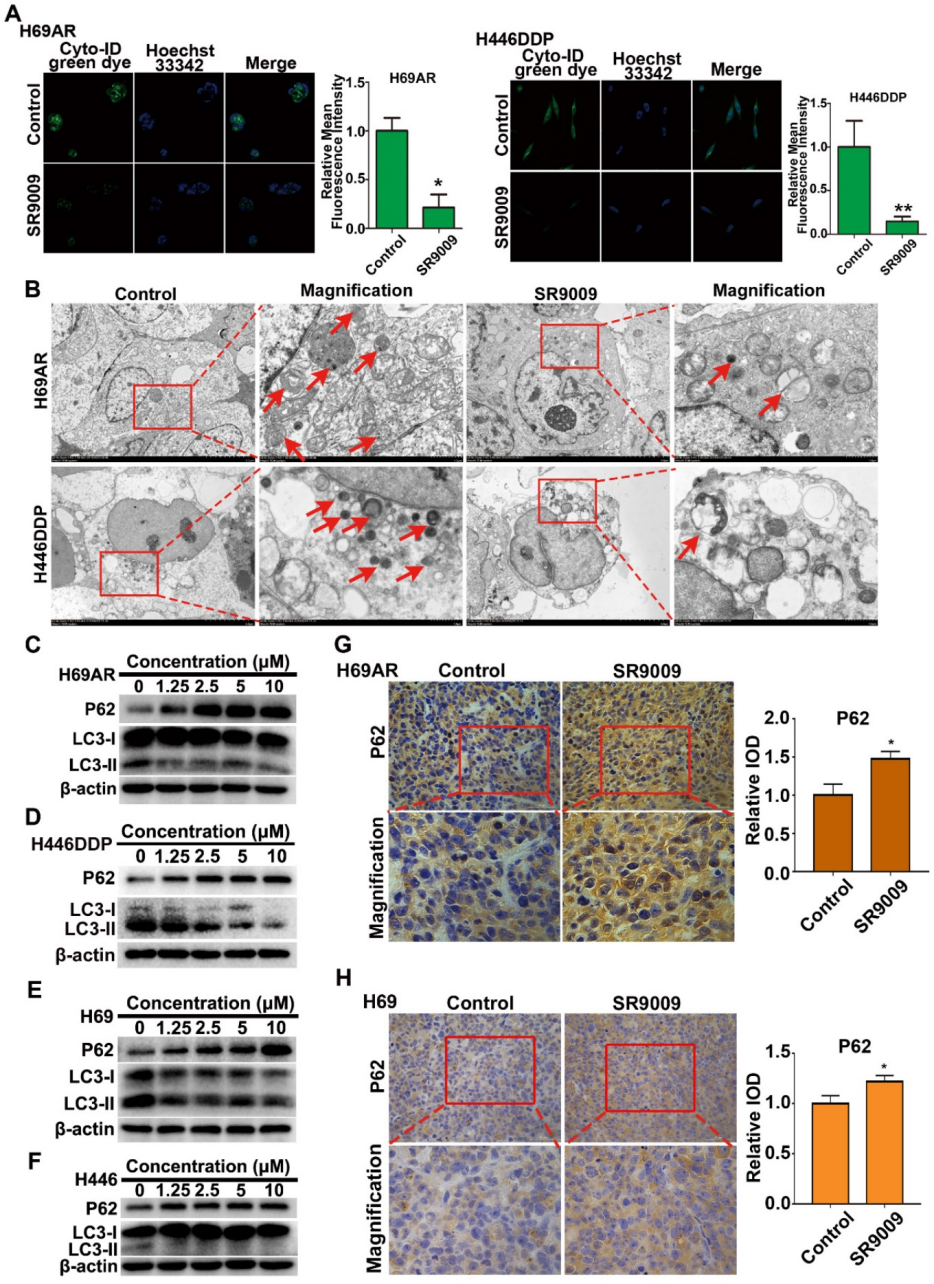
** SR9009 inhibited autophagy in SCLC cells. (A)** After stained with Cyto-ID Green dye, the formation of autophagic vesicles in H69AR and H446DDP cells treated with or without SR9009 for 48 h was detected by laser confocal microscopy. Quantification of autophagic vesicles (Cyto-ID) in cells were measured by the ImageJ software (n = 3, means ± S.D.). Confocal micrographs were taken at × 40. **(B)** H69AR and H446DDP cells were incubated with or without SR9009 for 48 h and TEM was employed to detect the autophagosomes, and the magnified view of the electron photomicrograph exhibited autophagosomes. Arrows, autophagosomes. A typical result from 3 independent experiments is presented. **(C and D)** H69AR and H446DDP cells were incubated with different concentrations of SR9009 for 48 h, then autophagy associate protein LC3-I/II and P62 was detected by western blot analysis. **(E and F)** H69 and H446 cells were incubated with different concentrations of SR9009 for 48 h, then LC3-I/II and P62 was detected by western blot analysis. **(G and H)** Representative IHC staining of autophagy associate protein P62 in subcutaneous xenografts of SR9009-treated group or vehicle group.

We initially analyzed basal autophagy activity in two pairs of SCLC cells. Consistent with previous reports 25, H69AR and H446DDP cells exhibited relatively high levels of autophagy (Figure S4A and B). Thus, to more directly verify the autophagy-inhibiting effect of the REV-ERBα agonist SR9009, we measured autophagy activity in H69AR and H446DDP cells treated with SR9009. Autophagosomes were evaluated using laser confocal microscopy, transmission electron microscopy (TEM) and western blotting in our study. The formation of autophagic vesicles was decreased in SR9009-treated cells, as detected by laser confocal microscopy and TEM (Figure 7A and B). Then, we examined the protein expression levels of LC3 and P62, two markers of autophagy, by western blotting. After treatment with SR9009, the expression level of the autophagy marker LC3-II was decreased, and P62 protein accumulated in a concentration-dependent manner (Figure 7C and D). A similar result was observed in H69 and H446 cells (Figure 7E and F). In addition, we examined the expression of P62 in the abovementioned SCLC subcutaneous tumor model. Immunohistochemical analysis showed hyperaccumulation of P62 in the SR9009-treated group (Figure 7G and H).

Autophagy is a dynamic process that begins with nucleation, which is followed by autophagosome formation, autophagosome-lysosome fusion and, finally, degradation of cellular contents in lysosomes 26. We sought to determine the stage of autophagy blocked by the agonist SR9009. Chloroquine (CQ) is an autophagy inhibitor that interrupts the fusion of autophagosomes to lysosomes, leading to the accumulation of autophagosomes. We compared the differential effects on autophagy of SR9009 and chloroquine and found that SR9009 reduced autophagosome formation, suggesting that SR9009 blocked autophagy at an early stage (Figure S5A and B).

### The antitumor effect of SR9009 was mediated via the inhibition of REV-ERBα-mediated autophagy

**Figure 8:**
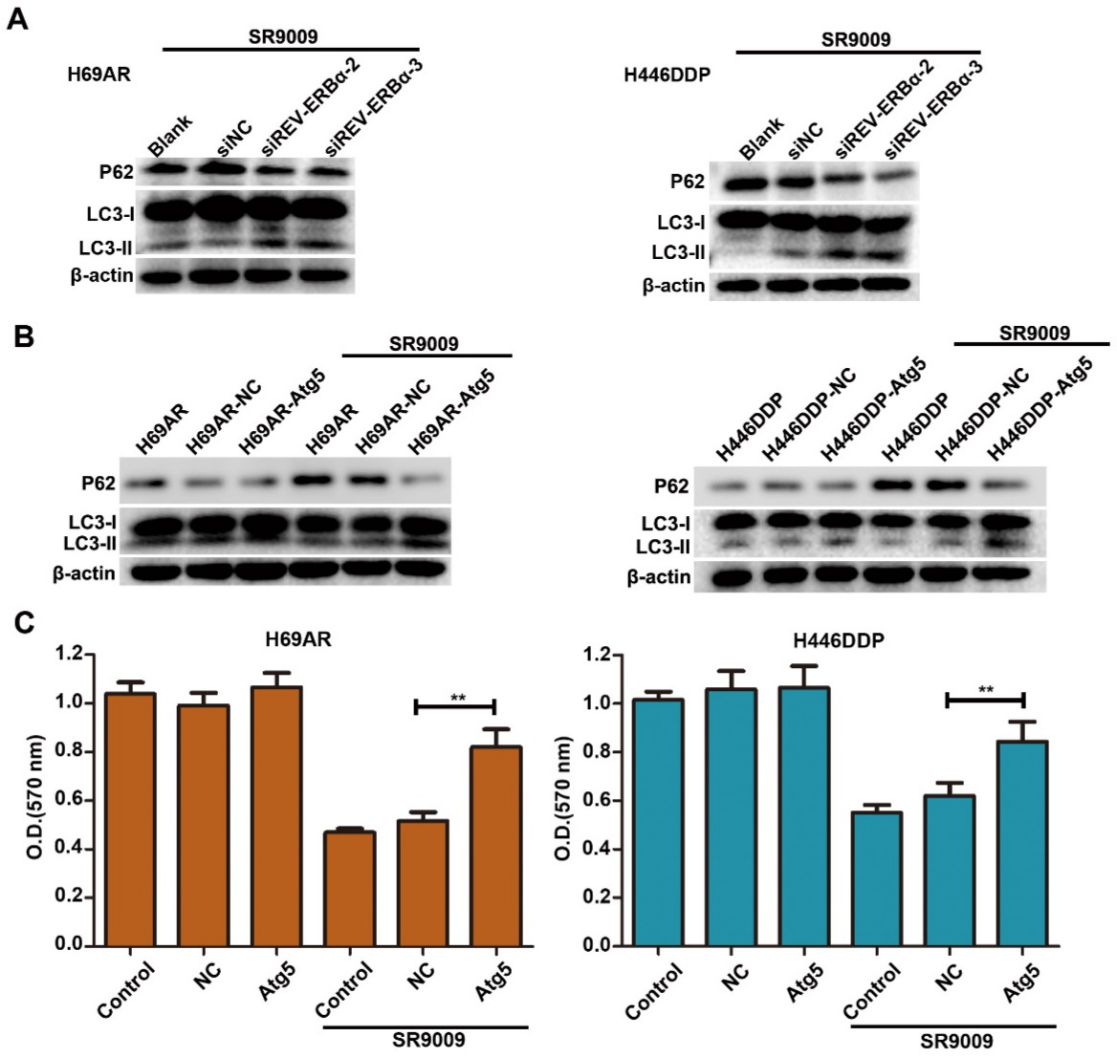
** The cytotoxicity effect of SR9009 was mediated by REV-ERBα autophagy inhibition. (A)** H69AR and H446DDP cells transfected with si-REV-ERBα or negative control vector were incubated with SR9009 for 48 h. The protein expression of autophagy associate protein LC3-I/II and P62 was measured by western blot analysis. **(B)** LC3II/I and P62 expression were detected by western blot analysis, after transfected with a plasmid encoding Atg5 or the corresponding negative control vector. (SR9009 treatment in H69AR and H446DDP cells, 10 µm). **(C)** CCK-8 assays showed the effect of upregulation of Atg5 on cytotoxicity induced by SR9009 (SR9009 treatment in H69AR and H446DDP cells, 10 µm). The data are presented as means ± SD of three independent experiments. **P < 0.01.

Our results showed that SR9009-induced cytotoxicity was markedly impaired by REV-ERBα knockdown (Figure 5G and H). As autophagy activity was potently inhibited by treatment for SCLC, we sought to determine whether autophagy regulated by REV-ERBα is involved in the antitumor mechanism of SR9009 in SCLC. Knockdown of REV-ERBα simultaneously diminished the autophagy-inhibiting effect of SR9009 (Figure 8A). Furthermore, after transfected with a plasmid encoding Atg5 or the corresponding negative control vector (Figure S6), overexpression of the core autophagy gene Atg5 restored the autophagy activity suppressed by SR9009 and abrogated the induction of cytotoxicity in SCLC cells (Figure 8B and C). Collectively, these data reveal that SR9009 induces cytotoxicity by negatively regulating REV-ERBα-mediated autophagy.

## Discussion

Despite the rapid advances in cancer therapies such as immunotherapy or molecular therapies targeting the epidermal growth factor receptor, the overall survival of SCLC has not significantly improved 27,28. The standard first-line therapy for SCLC is still CDDP combined with VP-16; however, chemoresistance develops rapidly and is followed by significant disease relapse 29. Therefore, it is urgent to develop a novel and more effective therapeutic approach. Circadian clock proteins coordinate gene expression and protein translation in human growth, metabolism and diseases 30,31. REV-ERBs are key circadian clock components that play an inhibitory role in many processes, including tumorigenesis, and SR9009 has been demonstrated to be a potent pharmacological agonist of REV-ERBs. In this article, we demonstrated for the first time that SR9009 exerts cytotoxicity effects in both chemosensitive and chemoresistant cell lines and animal models. In addition, we demonstrated that pretreatment with SR9009 could sensitize the resistant cells to chemotherapy. These founding extended the applications of SR9009 in the treatment of SCLC.

To further understand the antitumor mechanism of SR9009, we investigated its targets, REV-ERBα and REV-ERBβ. Our study showed that SCLC cells express fairly high protein levels of REV-ERBα compared to normal lung cells. These SCLC cells in our experiments express a very low level of REV-ERBβ and therefore they might not be the ideal to test whether REV-ERBβ is driving the anti-SLCL effects of SR9009. Then, the application of siRNAs to downregulate REV-ERBα in SCLC cell lines provided adequate data to support our claims that REV-ERBα protein activation was involved in the antitumor effect of SR9009. This effect might result because REV-ERBs are present primarily in the form of the REV-ERBα protein in SCLC.

Then, we applied ChIP-seq assays to screen molecules downstream of REV-ERBα and determined that the autophagy gene Atg5, as a downstream signal, was negatively regulated by REV-ERBα. Thus, REV-ERBα might modulate the growth of SCLC cells by regulating autophagy.

Multiple autophagy genes, such as Atg5, Atg7, Atg12, and LC3 (Atg8), have been reported to play key roles at various stages of autophagosome formation 32,33. Atg5 is a key protein involved in phagophore membrane elongation during autophagy and can associate with Atg12 to form covalent and constitutive Atg5-Atg12 conjugates 34. Upon the binding of the Atg5-Atg12 conjugate to Atg16, Atg5 regains its ability to associate with membranes. Phagophore elongation requires the incorporation of phosphatidylethanolamine (PE)-lipidated LC3 (Atg8) whose formation is facilitated by the Atg5-Atg12 conjugate and the Atg5-Atg12/Atg16 complex 35. The phagophore elongates until its membranes fuse, thus generating an autophagosome, which eventually fuses with a lysosome to form an autolysosome, where resident lysosomal hydrolases degrade the cargo 36,37. In this study, we found that targeting REV-ERBα with SR9009 negatively modulates autophagy through interaction with Atg5 in SCLC cells. This result suggested that the REV-ERBα agonist SR9009 might cause autophagy defects by repressing the Atg5 gene, leading to an anti-SCLC effect.

We identified that autophagy activity was markedly inhibited by SR9009 in SCLC. Many studies have revealed that cancer cells satisfy their high nutritional demands through complex compensation responses that involve elevated activation of autophagy, which is necessary for tumor cell survival. Accordingly, specific suppression of autophagy is a promising therapeutic strategy. Hence, it is critical to elucidate the effect of negatively regulating REV-ERB-mediated autophagy in SR9009 therapy for SCLC. Our experimental data indicated that knockdown of REV-ERBα or overexpression of the core autophagy gene Atg5 diminished the autophagy-inhibiting effect of SR9009. In addition, the induced cytotoxicity was significantly decreased in SCLC cells. Taken together, these findings indicate that SR9009 causes autophagy defects in order to impair SCLC growth via REV-ERBα-mediated downregulation of the core autophagy gene Atg 5.

## Conclusions

In summary, our results demonstrated for the first time that SR9009 exhibits an antitumor effect in SCLC. We identified that REV-ERBα was involved in the antitumor effect of SR9009 in SCLC, while REV-ERBα directly downregulates the key autophagy gene Atg5. Then, autophagy inhibition was assessed in SR9009-treated cells. Further research revealed that the antitumor effect of SR9009 is attributed to autophagy inhibition, which was mediated by direct downregulation of the core autophagy gene Atg5 by REV-ERBα. Therefore, we provided new insights indicating that pharmacological modulation of circadian clock components via SR9009 is a novel and promising therapeutic approach for SCLC and elucidated the relationship between circadian clock components and autophagy activity, which may contribute to the discovery of new potentially useful targets for SCLC therapy.

## Materials and Methods

### Cell lines

The human SCLC cell lines H69 and H446 and the chemoresistant cell line H69AR were purchased from the American Type Culture Collection (ATCC). The other drug-resistant subline, H446DDP, was established in our laboratory by culturing H446 cells in cisplatin and is described in our previous study 38. These cells were grown in RPMI 1640 medium supplemented with 10% fetal bovine serum and 1% penicillin/streptomycin and were incubated at 37°C in 5% CO2.

### Reagents

SR9009 was purchased from MedChemExpress (NJ, USA). Cyto-ID Green dye was purchased from Enzo Life Sciences, Inc. (Farmingdale, NY, USA). LysoTracker was purchased from Invitrogen (San Diego, CA, USA). The primary antibodies, including antibodies against β-actin, LC3B, SQSTM1/p62, cleaved caspase 3 and PARP, were obtained from Cell Signaling Technology (Danvers, MA, USA). The secondary antibodies were purchased from MR Biotech (Shanghai, China). The lysosomal inhibitor chloroquine was purchased from Sigma-Aldrich (St. Louis, MO, USA). Z-VAD-fmk was obtained from the Beyotime Institute of Biotechnology (Haimen, Jiangsu Province, China).

### Cell transfection

Cells were transiently transfected with validated siRNAs targeting REV-ERB and with the corresponding negative control siRNA (Suzhou GenePharma Co., Ltd., Jiangsu Province, China). Cells were mock transfected or transfected with siRNAs using Lipofectamine 3000 (L3000015, Invitrogen) for 48 h, and the knockdown efficiencies of the siRNAs were determined by western blot analysis.

### Transwell assay, and wound-healing assay

Transwell assay and wound-healing assay were performed as previously described 39.

### Apoptosis assay

Immunostaining for cleaved caspase 3 (Cell Signaling Technology, No. 9664, 1:200) was performed to evaluate apoptosis, and the TUNEL assay was performed using an In Situ Cell Death Detection Kit, Fluorescein (Roche) according to the manufacturer's protocol. Apoptosis was analyzed with a fluorescence microscope (Biological Research Microscope, Leica DMi8-M, Germany) equipped with a digital camera.

### Immunoblot analysis

SCLC cells were collected and washed with cold PBS and were then incubated at 0°C for approximately 30 min in Cell Lysis Buffer. After the protein concentration was determined with a BCA protein assay, equal quantities of protein were separated by SDS-PAGE and subsequently transferred to PVDF membranes. Membranes were blocked with 5% bovine serum albumin powder in Tris-buffered saline with Tween 20 (TBST). Then, membranes were incubated first with a primary antibody overnight at 4°C and then with a secondary horseradish peroxidase-conjugated antibody for 1.5 h. Finally, signals were detected with enhanced chemiluminescence reagents.

### TEM

SCLC cells were fixed with 2.5% glutaraldehyde containing 0.1 mol/L sodium cacodylate and treated with 1% osmium tetroxide. Samples were embedded in araldite, cut into thin sections, and stained with uranyl acetate and lead citrate. Samples were examined with a JEM 1400 transmission electron microscope (JEOL, Inc., Peabody, MA, USA) at 80 kV.

### Confocal microscopy

Cells were treated with or without SR9009 at the indicated concentrations and were then stained with an autophagy detection kit, Cyto-ID Green dye and LysoTracker Red. All samples were visualized by laser confocal microscopy (Carl Zeiss LSM710, Carl Zeiss, Oberkochen, Germany).

### Immunohistochemistry (IHC)

Tissue samples from the subcutaneous SCLC tumor models were fixed in 4% paraformaldehyde and embedded in paraffin blocks. Four-micron-thick sections were cut and analyzed for SQSTM1/P62 protein expression. Slides were incubated first with an anti-SQSTM1/P62 antibody at 4°C overnight and then with the secondary antibodies for 2 h. The results were visualized with the EnVision peroxidase system (Dako).

### Chromatin immunoprecipitation with sequencing data analysis

Peak calling was performed using model-based analysis of chromatin immunoprecipitation with sequencing (ChIP-seq) (MACS) and the false discovery rate as ≤0.05 40.

### Chromatin immunoprecipitation quantitative PCR (qPCR) assay

ChIP assays were conducted as previously described 41. An anti-REV-ERBα antibody (Cell Signaling Technology, No. 13418) was used. Immunoprecipitated DNA extracted from SCLC cells was analyzed by qPCR.

### *In vivo* study

The BALB/c nude mice used in this study were purchased from the Experimental Animal Center of Southern Medical University (Guangzhou, China) and maintained under specific pathogen-free conditions at Southern Medical University. SCLC cells were harvested and suspended in culture medium, and 1 × 10^7^ cells were subcutaneously injected to establish the SCLC xenograft model. When tumors reached an average size of 100 mm^3^, mice were randomly divided into four groups. Then, SR9009 (50 mg/kg) was administered intraperitoneally once every two days. Mice were intraperitoneally injected with physiological saline containing chemotherapeutics or physiological saline alone as a control. Mice received intraperitoneal injections of etoposide (4 mg/kg) once every 2 days and cisplatin (2 mg/kg) once every 8 days. The tumor sizes were measured using a caliper, and the volumes were calculated according to the following standard formula: V = (length × width^2^/2).

### Statistical analysis

The data were analyzed with GraphPad Prism 7 (GraphPad Software, Inc., La Jolla, CA, USA) and are presented as the means ± S.Ds. Comparisons between two groups were performed using Student's t-tests or one-way ANOVA. Differences with *P*-values of less than 0.05 were considered statistically significant. *P*-values of < 0.05 and < 0.01 are indicated with * and **, respectively.

## Supplementary Material

Supplementary figures.

## Abbreviations

SCLC: small cell lung cancer
ChIP: chromatin immunoprecipitation
IHC: immunohistochemistry
CCK-8: cell counting kit-8 assay
Cl.Casp.3: cleaved caspase 3
Ulk1: unc-51 like autophagy activating kinase 1
Atg5: autophagy protein 5
TEM: transmission electron microscopy
Bmal1: brain and muscle aryl hydrocarbon receptor nuclear translocator (ARNT)-like 1
Clock: clock circadian regulator
