# Supplementary material for: SR9009 induces a REV-ERB dependent anti-small-cell lung cancer effect through inhibition of autophagy
Source: Theranostics. 2020 Mar 15;10(10):4466–80. doi: 10.7150/thno.42478 (PMC7150483; doi:10.7150/thno.42478)
Supplement: Supplementary file 1 — Supplementary figures. [file thnov10p4466s1.pdf]

SR9009 induces a REV-ERB dependent anti-small-cell lung cancer effect through inhibition of autophagy

Weitao Shen[1,#], Wei Zhang[1,#], Weilin Ye[1#], Haihong Wang[2], Qingxi Zhang[3], Jie Shen[1], Qingsha Hong[4], Xiang Li[5], Ge Wen[6], Ting Wei[1,*], Jian Zhang[1,*]

Supplementary Materials

Supplementary Figures

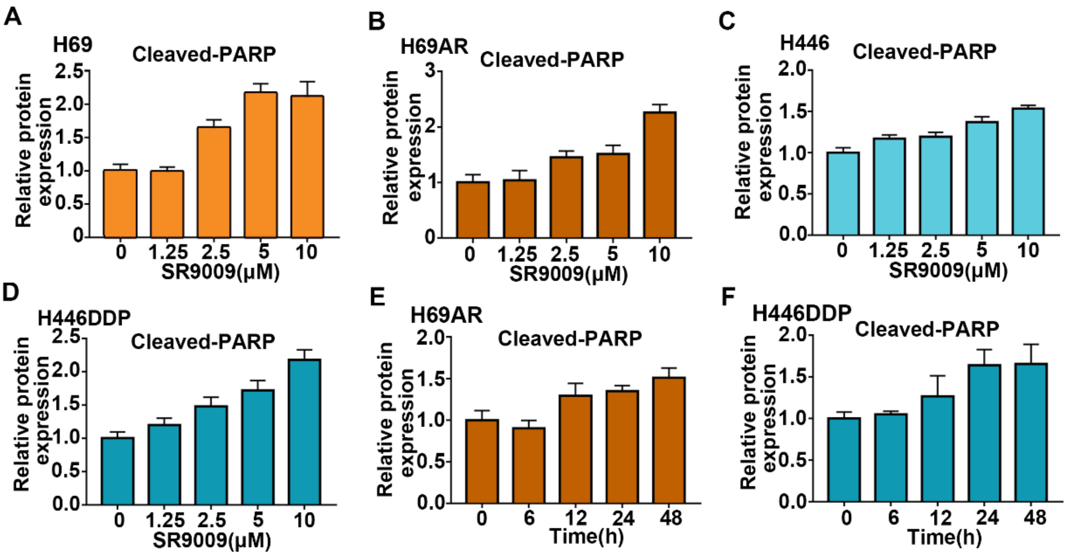

Figure S1

SR9009 induced remarkable apoptosis in SCLC cells. (A-F) Statistics of the Cleaved-PARP expression in Figure 2A-F. Densitometric values were quantified using the ImageJ software and normalized to control. The values of control were set to 1. The data are presented as means ± SD of 3 independent experiments.

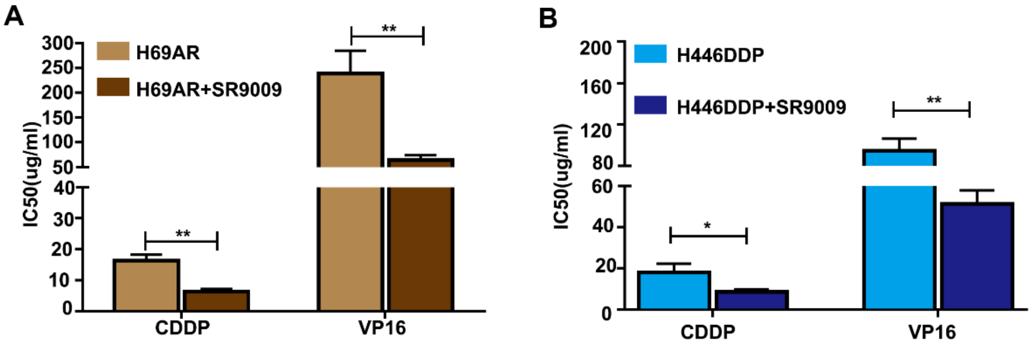

Figure S2

Pretreatment with SR9009 could sensitize the resistant cells to chemotherapy. (A and B) CCK-8 assays showed that pretreatment with SR9009 decreased the IC50 values to chemotherapeutic agents (CDDP or VP-16) of chemoresistant SCLC cells. The data are presented as means ± SD of three independent experiments. **P* < 0.05; ***P* < 0.01.

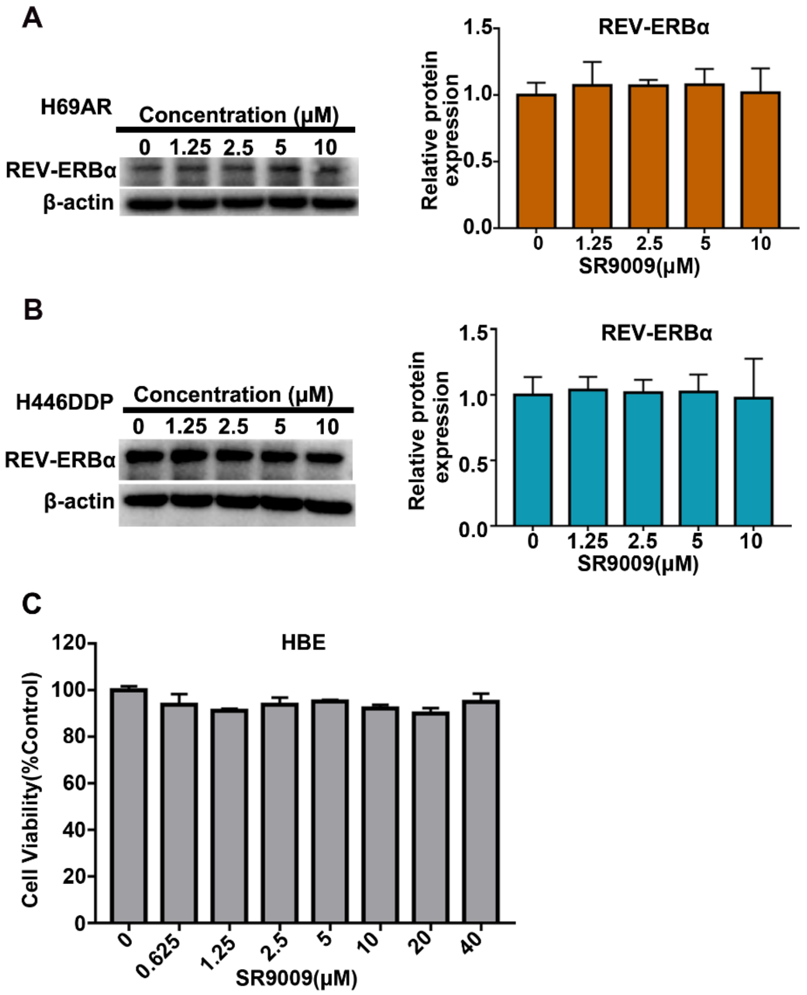

Figure S3

REV-ERBα was involved in the antitumor effect of SR9009 in SCLC. (A and B) H69AR and H446DDP cells were dose dependently treated with SR9009 for 48 h, and the protein expression of REV-ERBα was analyzed. Densitometric values were quantified using the ImageJ software and normalized to control. The values of control were set to 1. The data are presented as means ± SD of 3 independent experiments. (C) Normal human bronchial epithelial (HBE) cells were incubated for 72 h in different concentrations of SR9009. The data are presented as means ± S.D. of three independent experiments.

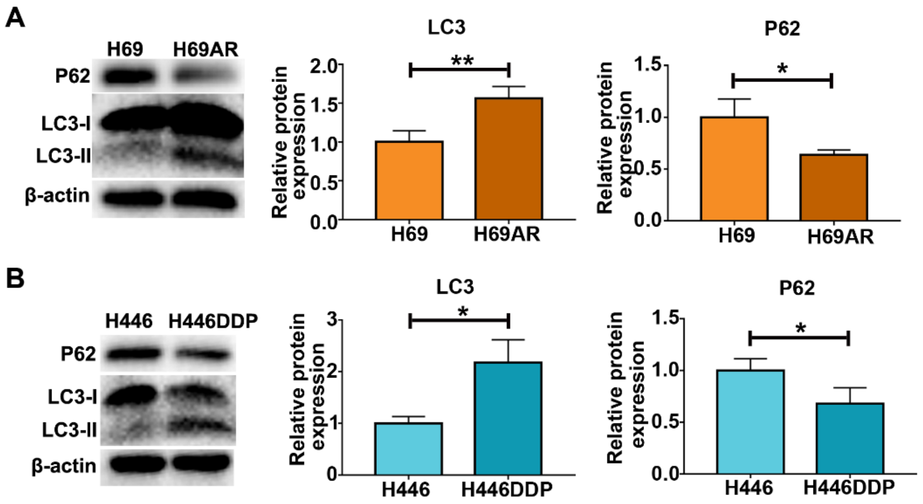

Figure S4

H69AR and H446DDP cells exhibited relatively high levels of autophagy. (A and B) Western blot analysis of LC3II/I and p62 expression in two pairs of chemosensitive and chemoresistant SCLC cell lines. Densitometric values were quantified using the ImageJ software and normalized to control. The values of control were set to 1. The data are presented as means ± SD of 3 independent experiments. $*P < 0.05$; $**P < 0.01$.

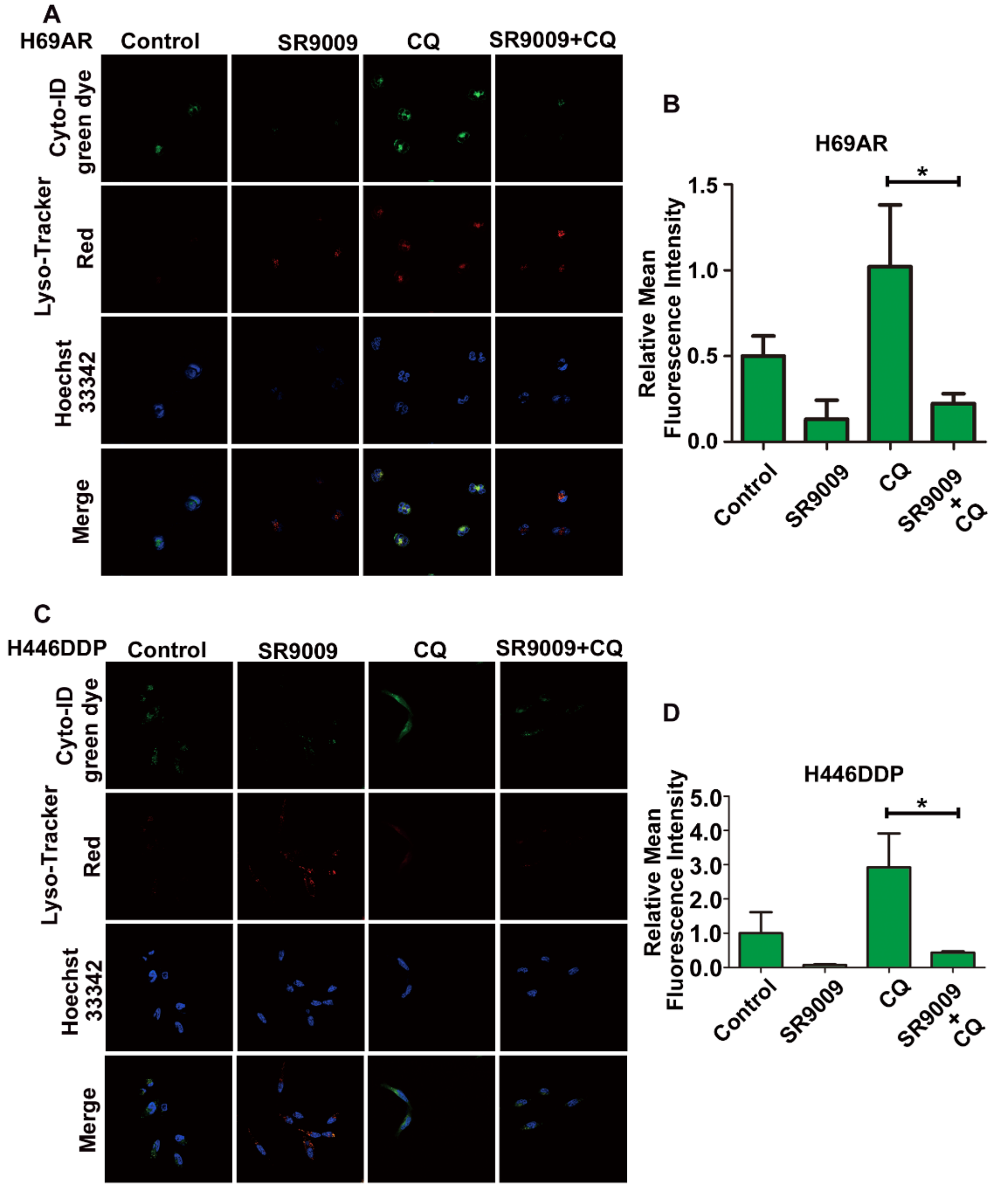

Figure S5

Autophagic flux was detected by confocal microscopy. (A and C) SR9009 blocked autophagy, which resulted in reduced autophagic flux. (B and D) Quantification of autophagic vesicles (Cyto-ID). The data are presented as means ± SD of 3 independent

experiments.*$P < 0.05$.

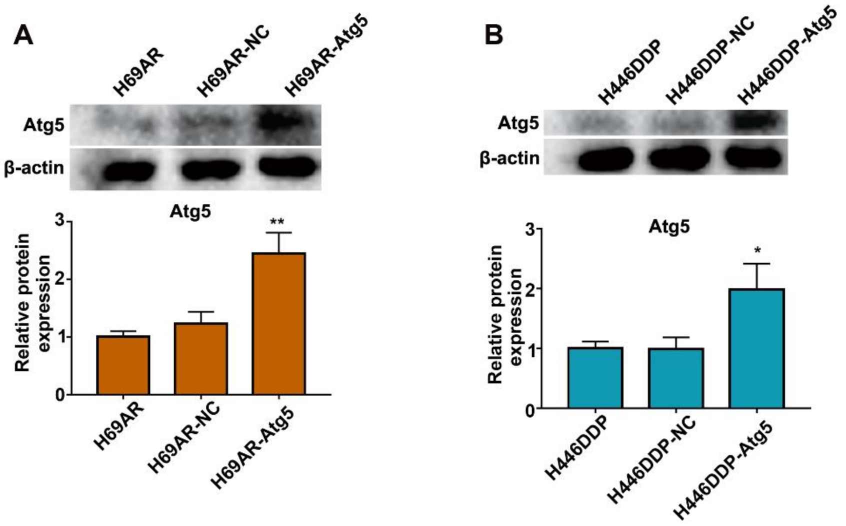

Figure S6

Western blot analysis of Atg5 in cells transfected with a plasmid encoding Atg5 or the corresponding negative control vector. Densitometric values were quantified using the ImageJ software and normalized to control. The values of control were set to 1. The data are presented as means ± SD of 3 independent experiments. *$P < 0.05$; **$P < 0.01$.
